# Wealth, mother's education and physical access as determinants of retail sector net use in rural Kenya

**DOI:** 10.1186/1475-2875-5-5

**Published:** 2006-01-26

**Authors:** Abdisalan M Noor, Judith A Omumbo, Abdinasir A Amin, Dejan Zurovac, Robert W Snow

**Affiliations:** 1 Malaria Public Health & Epidemiology Group, Centre for Geographic Medicine, Kenya Medical Research Institute, P.O. Box 43640, 00100, Nairobi, Kenya; 2 Centre for Tropical Medicine, John Radcliffe Hospital, University of Oxford, UK

## Abstract

**Background:**

Insecticide-treated bed nets (ITN) provide real hope for the reduction of the malaria burden across Africa. Understanding factors that determine access to ITN is crucial to debates surrounding the optimal delivery systems. The influence of homestead wealth on use of nets purchased from the retail sector is well documented, however, the competing influence of mother's education and physical access to net providers is less well understood.

**Methods:**

Between December 2004 and January 2005, a random sample of 72 rural communities was selected across four Kenyan districts. Demographic, assets, education and net use data were collected at homestead, mother and child (aged < 5 years) levels. An assets-based wealth index was developed using principal components analysis, travel time to net sources was modelled using geographic information systems, and factors influencing the use of retail sector nets explored using a multivariable logistic regression model.

**Results:**

Homestead heads and guardians of 3,755 children < 5 years of age were interviewed. Approximately 15% (562) of children slept under a net the night before the interview; 58% (327) of the nets used were purchased from the retail sector. Homestead wealth (adjusted OR = 10.17, 95% CI = 5.45–18.98), travel time to nearest market centres (adjusted OR = 0.51, 95% CI = 0.37–0.72) and mother's education (adjusted OR = 2.92, 95% CI = 1.93–4.41) were significantly associated with use of retail sector nets by children aged less than 5 years.

**Conclusion:**

Approaches to promoting access to nets through the retail sector disadvantage poor and remote communities where mothers are less well educated.

## Introduction

Insecticide treated bed nets (ITN) have been shown to provide significant protection against early childhood mortality under a range of malaria settings in Africa [1], reduce the incidence of clinical malaria and anaemia in young children [1,2] and are regarded as a cost-effective public health intervention for low income countries [3]. African Heads of State, as part of the Roll Back Malaria (RBM) initiative, agreed that they would ensure that 60% of at-risk populations in their countries would have access to ITN by 2005 [4]. Since the Abuja summit, donor support to country national malaria control programmes or their non-governmental organisations (NGO) partners has substantially increased to support the adoption of ITN policies and programme implementation [5]. Despite the overwhelming scientific evidence in support of the public health impact likely to result from widespread use of ITN, the political commitment and the increasing financial resources to support delivery, coverage among vulnerable young children remained poor in 2003 [5].

Factors that determine a community's use of nets should provide insight into why net coverage remains low even in countries where significant financial investments have been committed to expand coverage. Studies have shown that in most communities, net use is lowest among the poorest [6,7]. The role of education and distance to net providers has been less well documented. In this paper, the combined effects of proximate determinants of wealth, mother's education and physical access to markets on the use of nets purchased from the retail sector among rural children under five years of age in four districts in Kenya are examined.

## Methods

### The Kenya context

The Kenyan Ministry of Health (MoH) developed an ITN strategy paper in 2000, which spelt out the government's intentions for scaling up the use of ITN nationwide [8]. This was incorporated into the Kenyan National Malaria Strategy (NMS) as one of the four principal pillars of malaria prevention and control [9]. In 2001, Population Services International (PSI) launched a nationwide ITN social marketing programme with funding from the British Department for International Development (DFID). The aim of the project was to establish a "net culture" in the country through extensive marketing and education. Information campaigns were undertaken to increase basic knowledge on the causes of malaria and means of prevention, emphasizing the vulnerability of pregnant women and children under five years of age [10]. A rural kiosk-based distribution system was established to increase subsidised net availability run in parallel with joint programs to sell nets through other NGOs.

### Study area

The study was carried out in four districts purposively sampled by the MoH to provide detailed longitudinal information on coverage of interventions proposed as part of the NMS [11]. The study districts have been described elsewhere in detail [12,13] and represent the broad categories of malaria transmission across Kenya [14]. They include Kwale district on the coast with seasonal, high intensity malaria transmission; Bondo on the shores of Lake Victoria with high intensity perennial transmission; Kisii Central and Gucha districts (Greater Kisii) representing the low seasonal transmission conditions of the Western highlands; and Makueni district, a semi-arid area with acutely seasonal, low malaria transmission.

A geographic information system (GIS) was developed for each district reflecting high spatial resolution enumeration area (EA) population data, roads and footpaths, rivers, forests, gazetted areas and a 100 m Digital Elevation Model (DEM). In addition all market centres were mapped using Global Positioning Systems (GPS) [13,15]. An EA was defined by the Central Bureau of Statistics (CBS) during the 1999 national census as an area of about 500 people or 100 homesteads [16]. The GIS data were all stored in ArcView GIS 3.2 (ESRI Inc., New York, USA) raster format.

### Sampling and homestead surveys

A larger randomly sampled community survey undertaken in 2001, reported elsewhere [12,17] provided the sampling frame for the present study. EA sampling was done to ensure that there was enough power and precision to detect changes in net use over time among rural communities. The study was based on a cross-sectional survey with the unit of analysis being a child under the age of 5 years residing in a homestead within a randomly selected rural EA cluster. In each district a total of 18 rural EAs were randomly selected. All homesteads within the sampled EAs were surveyed. Questionnaires were translated into the primary language of each district and back-translated into English by independent assessors fluent in each language to identify and correct ambiguities in translation and meaning. Field staff were recruited at each district and trained over three days in the survey instruments, how to approach homesteads and check data in the field. Fieldwork began during November 2004 to coincide with the short rainy season in each district. Field staff used GIS generated district and EA maps to locate each homestead within selected EA. All members of the homestead were enumerated and assigned a unique identification number. Homestead heads or responsible members of the homestead were interviewed as part of the survey related to homestead socio-demographic characteristics, assets and other wealth indicators (Table 5) and malaria prevention practices. Also captured was information on homestead head's education and main source of income. Mothers or guardians of all children under the age of five were asked specific questions related to the child's use of nets, whether the nets had been treated with insecticides and net sources. Details of the mother's age, marital status, educational attainment and source of income were recorded.

Data entry and storage was undertaken using MS Access (Microsoft, Redmond, USA), through customised data entry screens with in-built range and consistency checks. Descriptive summaries of all information were generated using STATA version 8.2 (Statacorp 2003, College Station, USA) and MS Excel 2002 (Microsoft, Redmond, USA). All information specific to the EA, homestead, mother or guardian were linked to the relevant child through the use of a primary homestead identifier that was consistent across all data sets.

### Creating a homestead assets-based wealth index

Principal component analysis (PCA) was performed, using STATA, to construct a homestead wealth assets index from information on the broad range of indicators collected during the survey. PCA is a data reduction technique that provides a method of identifying, from a large set of variables, those that contain most of the information common to all. The first principal component often represents the linear index of these important variables with most information [6,18]. Quintile distributions were derived for each district separately based on this assets index. Quintile descriptions based on district-specific homestead wealth assets index were used to allow for aggregated wealth assets to be defined at the district level rather than across districts. The district effects were then adjusted for during the regression analysis.

### Travel time to market centres

GPS coordinates for homesteads and market centres were used to develop physical accessibility models. Most studies of physical access to health interventions use straight-line (Euclidean) distances between population and service locations [19,20]. Previous work has shown that this approach does not accurately reflect actual distances travelled and overestimates the extent of physical access to interventions especially in rural settings [21]. A travel time model was developed from the high-resolution spatial raster data on market centres, transport network, rivers, permanent water bodies, topography and land cover for four the study districts. Using a GIS algorithm based on the specifications of the Naismith-Langmuir rule for pedestrian movement [21,22] the model simulated the ease or difficulty of physical access to markets centres based on the presence of a road or footpath, changes in slope along the path and restriction caused by the presence of barriers such as rivers, forests or gazetted areas. This rule states that a walker can maintain a speed of 5 km/h on flat road; 2.5 km/h on level ground off-road; 1 hour added for every 600 m of ascent; 10 minutes subtracted for each 300 m moderate descents (-5° to -12°); and 10 minutes added for each 300 m steep descent (steeper than -12°). The algorithm used an iterative region-growing approach in which each pixel containing a market centre was taken as a 'seed' pixel around which regions of travel time pixels were grown. Only the fastest route to a given pixel was used to calculate travel time to the market centre. Only pedestrian motion was modelled as majority of people in rural homesteads of the study districts walked to market centres. Travel time to the nearest market centre was assigned to each homestead.

### Statistical analyses of predictors of use of nets purchased from the retail sector

The study set out to look at predictors of use of nets by children <5 years of age obtained from the retail sector as these represent the principal sources of nets during the periods leading up to the time of the survey. Therefore, children who used nets purchased from the retail sector were compared to those who did not use nets. Children who used nets from sources other than the retail sector were excluded from the regression analysis. First, a univariate regression analysis was performed to identify which of the predictor variables were significant to the outcome measure, i.e. use of nets purchased from retail sector by children. These predictors defined homestead, mother and child characteristics. In the univariate analysis the odds ratio (OR), *p*-value and 95% confidence interval (CI) for each factor's association with nets purchased from the retail sector were computed. Any factor with a *p*-value < 0.15 was considered to be a potentially important covariate of retail-net use. The factors examined were: homestead wealth assets index; gender of homestead head; homestead demographics; travel time to the nearest market centre; homestead use of insecticide residual spraying (IRS); whether any homestead member attended public malaria awareness meeting (*baraza*) or owned printed education materials on malaria; whether mother could read; mother's education level (no education, primary incomplete, primary complete and secondary and above); mother's main source of income; mother's marital status; whether mother was pregnant; age of the child; sex of the child; and child's ownership of a health card.

All variables meeting the entrance criteria were used to estimate a multivariable logistic regression model to identify their combined effect on the use of nets purchased from the retail sector among children. The model was fitted using the STATA *xtgee *command with an exchangeable working correlation matrix. This procedure uses generalized estimating equations (GEE) to account for the potential correlation of use of nets purchased from the retail sector among children seen in the same EA while accounting for the variability between clusters. Results for all districts were combined and clustering was defined at the primary sampling unit, the EA. All results were weighted for unequal probability of selection of EA within each district (weight = 1/probability of selecting an EA). Parameter estimates: OR; 95% CI; and P-values; were recorded for each predictor. All predictors were adjusted for the effect of the variation between districts in the multivariable analysis.

## Results

### Study participants

Seventy two rural EAs were sampled in the four districts, covering a total of 2,807 homesteads. Three homesteads refused to participate in the study while 104 had homestead heads or other responsible members away on visits during the survey (and after re-visits). Overall 2,700 homesteads were surveyed (a response rate of 96.2%) but completed questionnaires for five of them were lost, reducing the number of rural homesteads in the database to 2,695 with a total population of 24,020, including 3,755 children under the age of five years belonging to 2,603 mothers/guardians in 1,731 homesteads. A total of 562 (15.0%) children slept under a net the night before the interview. 327 (58.2%) slept under nets purchased from the retail sector of which 224 (68.5%) were socially marketed by PSI (Supanet^®^). 235 slept under nets obtained from a variety of other sources, including subsidised nets through antenatal clinics (ANC) (Table 1).

**Table 1:** Nets use and net sources among rural children in four districts in Kenya, 2004

	Bondo	Greater Kisii	Kwale	Makueni	Total
Number of children seen (a)	793	934	1,283	745	3,755
Children who slept under a net (%)	146 (18.4)	95 (10.1)	237 (18.5)	84 (11.3)	562 (15.0)
Source of nets					
Retail outlets (%)	70 (47.9)	62 (65.3)	119 (50.2)	76 (90.5)	327 (58.2)
Other (%) (b)*					
NGO/MOH Community pharmacy	5 (3.4)	26 (27.4)	51 (21.5)	1 (1.2)	83 (14.8)
MoH ANC health facility	6 (4.1)	4 (4.2)	64 (27.0)	4 (4.8)	78 (13.9)
Mission health facility	4 (2.7)	3 (3.2)	0 (0.0)	0 (0.0)	7 (1.3)
CDC/KEMRI programme	55 (37.7)	0 (0.0)	0 (0.0)	0 (0.0)	55 (9.8)
Net given as a gift	6 (4.1)	0 (0.0)	3 (1.3)	3 (3.6)	12 (2.1)
Total	76 (52.1)	33 (34.7)	118 (49.8)	8 (9.5)	235 (41.8)
Number of children included in the analysis (%) (a-b)	717 (90.4)	901 (96.5)	1,165 (90.1)	737 (98.9)	3,520 (93.7)
Net treatment					
Total nets treated (%) †	95 (65.1)	53 (55.8)	98 (41.4)	25 (29.8)	271 (82.9)
Total retail sector nets treated (%)‡	32 (45.7)	32 (51.6)	50 (42.0)	22 (28.9)	136 (41.6)
Types of retail sector nets					
PSI brand (SUPANET^®^) (%)	48 (68.6)	43 (69.4)	87 (73.1)	46 (60.5)	224 (68.5)
Other brands (%) **	22 (31.4)	19 (30.6)	32 (26.9)	30 (39.5)	103 (31.5)

* Children who slept under nets from these sources were eventually excluded from the analysis since the outcome measure was children's use of retail nets with reference to those who didn't use nets at all.

** Other brands include: Aggrevo; Magic Marble; Nettee; Didi's net; Western Kenya net; Mmbu net, Globe; DawaNet; 1CDC/KEMRI net; Ziggy's; Top; Xpress; 1^st ^Choice; Flag; Impact; Kingganet; or unbranded

† Percentages represent proportion of children who slept under nets treated in the six months prior to the survey out of all those who slept under a net the night before the interview

‡ Percentages represent the proportion of children who slept under nets purchased from the retail sector and treated in the six months prior to the survey out of all those who slept under retail nets the night before the interview

### Output of the PCA

Data on 46 asset indicators for the 2,657/2,965 homesteads were used in the PCA to construct homestead level wealth assets index (Table 5). There were differences in the set of indicators accounting for the biggest overall effect on the homestead wealth assets index between the districts. For example in Bondo, houses with stone/concrete walls increased the homestead wealth assets index the most (1.60) while in Greater Kisii occupants paying rent in Greater Kisii contributed the most to the index (3.09). The index assigned 15.4% of the children in the analysis to homesteads in the least poor quintile while 20.1% to those in the most poor quintile. Overall, use of nets purchased from the retail sector increased with homestead wealth and was significantly different between the top and lowest quintiles (least poor, 40.1%; most poor, 5.5%; χ^2 ^= 137.1, p < 0.0001). The univariate regression of use of nets purchased from the retail sector against homestead wealth assets index showed that the least poor children were more than five times more likely to use retail sector nets than the most poor, even after this was adjusted for the variation between districts (OR = 5.12, 95% CI = 3.38–7.97; adjusted OR = 5.30, 95% CI = 3.42–9.20) (Table 2).

**Table 2:** Variation of retail sector net use with homestead wealth assets index, mother's education and travel time (hours) to nearest market centres among rural children < 5 years of age in four districts in Kenya, 2004

	No nets (3193) *n *(%)	Retail sector nets (327) *n *(%)	**Odds ratio (OR) (95% CI)**^†^	**Adjusted OR (95% CI)**^‡^
**Homestead wealth assets index quintile**				
Most poor	690 (21.6)	18 (5.5)	1	1
Very poor	716 (22.4)	34 (10.4)	0.49 (0.25–0.95)	0.48 (0.25–0.95)
Poor	679 (21.3)	57 (17.4)	0.78 (0.51–1.21)*	0.78 (0.51–1.21)*
Less poor	644 (20.2)	84 (25.7)	1.19 (0.80–1.78)	1.19 (0.79–1.78)
Least poor	410 (12.8)	131(40.1)***	5.12 (3.38–7.97)***	5.30 (3.42–9.21)***
Not determined††	54 (1.7)	3 (0.9)	-	-
**Mother's education**				
No education	760 (23.8)	47 (14.4)	1	1
Primary incomplete	998 (31.3)	78 (23.9)	0.79 (0.55–1.14)	0.79 (0.56–1.10)
Primary complete	820 (25.7)	94 (28.7)	1.17 (0.79–1.73)	1.21 (0.81–1.82)
Secondary and above	582 (18.2)	107 (32.7)***	2.42 (1.64–3.57)***	2.92 (1.93–4.41)***
Not determined‡‡	33 (1.0)	1 (0.3)	-	-
**Travel time to nearest market centre (minutes)**				
Mean (min, max)	34.8 (1.2, 158.4)	24.0 (1.2, 138.0)		
0–24	1745 (54.7)	220 (67.3)	1	1
>24–48	683 (21.4)	77 (23.5)	1.32 (0.70–2.51)	1.28 (0.69–2.44)
>48	765 (23.9)	30 (9.2)***	0.25 (0.13–0.44)***	0.18 (0.09–0.38)***

*P < 0.05; ** *P *< 0.01; *** *P *< 0.001 derived from Pearson's χ^2 ^test of proportion and univariate logistic regression

† Parameter estimates (OR, CI and P-values) were obtained using logistic regression adjusted for clustering. Clusters were defined at the EA level and weighted (weight = 1/probability of selection of an EA) using STATA 8.2 (Statacorp, Inc. USA)

‡ Odds ratios and CI here have been adjusted for the effect of variation between districts

†† 57 children were from 38 homesteads whose wealth assets index could not be determined using PCA because information on one or more asset indicators was missing.

‡‡ Mother's education level could not be determined for 34 children belonging to 33 mothers

### Mother's level of education

Children's use of nets purchased from the retail sector was shown to be closely correlated with mother's education, with only 14.4% of children of uneducated mothers using nets compared with 32.7% of those whose mother's had education up to secondary level and above (χ^2 ^= 37.91, p < 0.0001). The univariate logistic regression of use of nets purchased from the retail sector with mother's education level (with and without adjusting for district effects) showed that children whose mothers were educated to secondary level or above were up to three times more likely to use nets purchased from the retail sector than were those whose mothers were not educated (OR = 2.42, 95% CI = 1.64–3.57; adjusted OR = 2.92, a95% CI = 1.93–4.41) (Table 2).

### Travel time to nearest market centres

Modelled pedestrian travel time to the nearest market centre was used as an indicator of access to sources of retail sector nets. The mean travel time to market centres for users of nets purchased from the retail sector was 24 minutes compared to 35 minutes for those who were without these nets (Table 2). Most of the children, (54.7% of those who did not use nets purchased from the retail sector and 67.3% of those who did), were within 24 minutes of the nearest market centre. There was a significant difference in use of nets purchased from the retail sector with travel time between children within 24 minutes of the nearest market centre and those 48 minutes or more (χ^2 ^= 37.85, p < 0.0001). The univariate logistic regression showed that each log unit increase in travel time resulted in a 75–82% decreased probability of using a net purchased from the retail sector (OR = 0.25, 95% CI = 0.13–0.44; adjusted OR = 0.18, 95% CI = 0.09–0.38) (Table 2).

### Predictors of net use

The 15 variables studied as potential predictors of net use were structured at different levels: community; homestead; mother; and child. Of these, three had univariate p values of ≥ 0.15 and were dropped from the multivariable analysis (Table 3). Two other variables (mother's main source of income and homestead use of Indoor Residual Spraying (IRS) were also dropped because the former was highly correlated with homestead wealth assets index and mother's education level while over 95% of the children lived in homesteads which did not use IRS. In the descriptive analysis, all the three key variables (homestead wealth assets index, mother's education and travel time to market centres) showed a significant association with use of nets purchased from the retail sector (Table 2). Mother's education, however, was found to be highly correlated with homestead wealth asset index and was excluded from the final model (Table 3). When the model was fitted with either mother's education level or homestead wealth asset index only, both predictors showed significant association with use of nets purchased from the retail sector (results not shown).

**Table 3:** Univariate statistical associations of factors with use of retail sector nets among rural children < 5 years of age in four districts of Kenya, 2004. These factors were excluded from the multivariable analysis

	Number (%) in each category				
	**No nets (*n *= 3193)**	**Retail sector nets (*n *= 327)**	**OR**	**95% CI**	**P-value**

**Homestead level predictors**					
Homestead used insecticide residual spraying*					
No	3064 (96.0)	300 (91.7)	Ref.		
Yes	119 (3.7)	27 (8.3)	2.36	1.29–4.30	0.005
Not determined	10 (0.3)				
**Homestead demography†**					
Population 15–44 yrs of age					
Mean (min, max	6.0 (0, 43)	4.9 (0, 43)	0.89	0.68–1.17	0.401
**Mother level predictors**					
**Mother's age†**					
Mean (min, max)	29.1 (13, 103)	28.8 (16, 95)	0.87	0.59–1.28	0.476
**Mother can read†**					
No	957 (30.3)	57 (17.7)			
Yes	2204 (69.7)	269 (82.3)	1.91	1.12–3.24	0.16
Mother's main source of income*					
Unemployed/economically inactive	445 (13.9)	41 (12.5)	1		
Unpaid working on family business/farm	2321 (72.7)	205 (62.7)	0.64	0.46–0.89	0.008
Works for pay/receives money from spouse or homestead members	427 (13.4)	81 (24.8)	1.68	1.17–2.42	0.005
**Child level predictors**					
**Child sex†**					
Female	1597 (50.0)	154 (47.1)			
Male	1596 (50.0)	173 (52.9)	1.01	0.86–1.18	0.928

* These predictors were excluded from the multivariable analysis because: most children were from homesteads that did not use IRS (95.8%); mother's main source of income was highly correlated with homestead SES and mother's education level

† These predictors were excluded from the multivariable analysis because they did not meet the entrance criteria (p ≤ 0.15)

Multivariable analysis of the remaining 9 variables showed a variety of associations with use of nets purchased from the retail sector (Table 4). All predictors were adjusted for the effect of the district in the multivariable regression. Homestead wealth assets index showed the strongest significant association with use of nets purchased from the retail sector. Children in the least poor quintile were 10 times more likely to use nets from this sector than those in the poorest quintile. Those in the intermediate quintiles were also between two to five times more likely to use nets purchased from the retail sector than children living in the poorest homesteads. Travel time to the nearest market centre (transformed using natural logarithms) remained strongly associated with use of nets purchased from the retail sector use even in the multivariable model, with every log unit increase in time resulting in 49% less likelihood of using these nets. The gender of the homestead head was not associated with use of nets purchased from the retail sector. The presence of older children of primary school going age (5–14 years) appeared to reduce the likelihood of use nets from the retail sector among younger children (adjusted OR = 0.68, 95% CI = 0.52–0.90). Surprisingly, attendance at public meetings or ownership of printed materials related to raising malaria awareness appeared to have a negative association with use of nets from the retail sector (adjusted OR = 0.70, 95% CI = 0.46–1.05), although this was only of borderline statistical significance (p = 0.087). Children of mothers who were married or unmarried but living with partners were significantly more likely to use nets purchased from the retail sector than those whose mothers were either single or widowed (adjusted OR = 2.74, 95% CI = 1.83–4.10). Children older than one year and those belonging to mothers who were pregnant at the time of the survey were 29% and 41% less likely to use nets purchased from the retail sector respectively. Children who owned an immunisation card were twice as likely to use nets purchased from the retail sector as those who did not have a card.

**Table 4:** Homestead, mother and child factors* associated with use of retail sector nets among rural children < 5 years of age in four districts in Kenya, 2004: multivariable analysis results

	Number (%) in each category				
	**No nets**	**Retail sector nets**	aOR**	a95% CI**	**P-value**

**Homestead level predictors**					
**Homestead wealth assets index quintile**					
Most poor	690 (21.6)	18 (5.5)	Ref.		
Very poor	716 (22.4)	34 (10.4)	2.33	1.07–5.08	0.033
Poor	679 (21.3)	57 (17.4)	3.05	1.45–6.40	0.003
Less poor	644 (20.2)	84 (25.7)	4.80	2.35–9.79	<0.0001
Least poor	410 (12.8)	131(40.1)	10.17	5.45–18.98	<0.0001
Not determined	54 (1.7)	3 (0.9)			
**Travel time to nearest market centre (hours)**					
Mean (min, max)	34.8 (1.2,158.4)	24.0 (1.2, 138.0)	0.51	0.37–0.72	<0.0001
**Homestead head sex**					
Female	248 (7.8)	13 (4.0)	Ref.		
Male	2945 (92.2)	314 (96.0)	1.43	0.94–2.16	0.093
**Homestead demographics‡**					
Population <5 yrs of age (mean (min, max))	3.2 (0, 16)	2.6 (1, 16)	0.86	0.57–1.31	0.485
Population 5–14 yrs of age (mean (min, max))	3.8 (0, 25)	2.8 (0, 21)	0.68	0.52–0.90	0.007
Population >44 yrs of age (mean (min, max))	1.6 (0, 8)	1.2 (0, 7)	0.84	0.60–1.18	0.314
**Homestead member attended *baraza*/owns printed materials**					
No	2287 (71.6)	261 (79.8)	Ref.		
Yes	906 (28.4)	66 (20.2)	0.70	0.46–1.05	0.087
**Mother level predictors**					
**Mother is pregnant**					
No	2690 (91.0)	303 (95.3)	Ref.		
Yes	266 (9.0)	15 (4.7)	0.59	0.36–0.97	0.037
**Mother's marital status**					
Not married	469 (14.9)	29 (14.3)	Ref.		
Married	2690 (85.1)	295 (85.7)	2.74	1.83–4.10	<0.0001
**Child level predictors**					
**Child age**					
<12 months	654 (20.5)	90 (27.5)	Ref.		
≥12–59 months	2539 (79.5)	237 (72.5)	0.71	0.54–0.92	0.011
**Ownership of immunisation card**					
No	581 (18.2)	37 (11.3)	Ref.		
Yes	2609 (81.8)	290 (88.7)	2.00	1.10–3.64	0.023

* Predictors whose multivariable results are listed in this table all had p < 0.15 in the univariate analysis

** aOR and a95% CI stand for adjusted odd ratios and adjusted 95% confidence intervals respectively. Adjustment was done for the effect of the districts

‡ Predictors under homestead demographics were transformed using natural logarithms because their probability density functions (pdf) showed that they were not normally distributed Ref. = reference level

## Discussion

The predominant national delivery model for nets between 2001 and 2004 in Kenya was the adoption of a social marketing through the retail sector. PSI was awarded over 22 million UK pounds for a five year programme beginning in 2001 aiming to achieve at least 60% ITN coverage within 5 years [10,23]. Our data suggest that this model of delivery had fallen a long way short of its proposed programme target and those set by the international RBM community in rural areas of four districts in Kenya. Only 15% of children in 72 randomly selected communities were protected by a net, of which 48% were treated with an insecticide during the previous six months. Comparisons with 2001 data from these same communities suggests that ITN coverage among children increased from 3% in 2000/1 (unpublished data) to 7% in 2004/5. In 2004/5 approximately 70% of nets obtained from the retail sector were those marketed by PSI. However, there is a clear unmet need. The present study explored reasons why children might not be protected by a net obtained from a heavily funded programme to promote access to nets in greater depth.

A number of traditional determinants of health service use (wealth and education) and a novel, but important variable (distance to retail sector providers) were used within a combined model to examine the independent contribution of these competing factors. Results show that the use of nets purchased from the retail sector was lowest among children from the poorest homesteads, with retail sector net use increasing monotonically with homestead wealth. Children of mothers with the highest education (secondary level and above) were twice as likely to use nets purchased from the retail sector compared to mothers who had no formal education. In addition, a child who lived in a homestead closer to a market centre was more likely to use nets purchased from the retail sector than those who lived at greater distances, with a unit increase in travel time reducing chances of using nets from the retail sector by almost half. Only 1.5% of children born to mothers with no formal education living in the poorest homesteads over 48 minutes from a market centre used a net socially marketed through the retail sector. This extreme example demonstrates the polarising nature of compounding risks of retail sector net distribution.

Other factors seem to influence the use of nets purchased from the retail sector. The presence of older children in the homestead seemed to potentially reduce the chances of children under the age of five using nets. A higher proportion of children of married mothers or those living with a partner used nets from the retail sector than those of single or widowed mothers. Ownership of an immunisation card also seemed to increase the child's chances of using a net from the retail sector. One could speculate that the demands put on already strained homestead resources by older children, e.g. for education, may reduce the chances of a homestead owning a net. However, more formative research is required to establish whether this is true and whether in the face of a newly established policy for universal free primary education in Kenya this might change. The majority of mother's looking after their children without the help of partners were self-employed (64%) and the time and economic constraints on single parents probably diminishes their capacity to procure nets from the retail sector for their children. The reasons why ownership of immunization card is associated with use of nets purchased from the retail sector are not intuitively obvious but might be a proxy for general health awareness. Interestingly, however, attendance at a malaria awareness meeting during the preceding 12 months or ownership of printed malaria materials was not significantly associated with use of nets purchased from the retail sector.

## Conclusion

Despite a large financial investment in social marketing through the retail sector, net coverage in rural, Kenyan communities remains low and unlikely to meet RBM or national targets set for 2005/6. The most disadvantaged are those children both economically and geographically vulnerable. Ensuring equitable access and coverage must remain a priority. Toward the end of 2004 PSI adapted their strategy of net promotion to include heavily subsidised nets delivered through routine antenatal service and immunization clinics. This should improve spatial accessibility and affordability of nets to a larger sector of the rural poor. The vulnerability analysis undertaken as part of the present study should provide a model of analysis in 2006 to determine whether geographical and wealth barriers are overcome through this new approach to net delivery.

## Authors' contributions

AMN was responsible for the conception and design of the study, collection, interpretation, analysis of the data and writing of the paper. JAO was contributed to the collection of data and revision of manuscript. AAA contributed to the interpretation of data and revision of manuscript.

DZ also contributed to the interpretation of data and revision of manuscript. RWS provided overall intellectual support, guidance to the work and commented on the manuscript.

**Table 5 - Appendix:** Summary of variables used in the computation homestead socio-economic status asset index from the first principal component

	Homestead wealth asset index			
**Variable**	**Bondo**	**Greater Kisii**	**Kwale**	**Makueni**

**Ownership of livestock**				
1. Number of cows	0.03643	0.00758	0.00102	0.05416
2. Number of shoats	0.02725	0.00068	0.00039	0.01281
3. Number of donkeys	0.06534	0.02683	-0.02545	0.238
4. Number of chickens	0.01076	0.00335	0.00152	0.01937
5. Number of ducks	0.03485	0.06381	0.01872	0.10848
**Homestead head (HH) education level**				
6. No education	0	0	0	-0.02969
7. Primary incomplete	0.15034	0.07051	0.26061	0
8. Primary complete	0.25688	0.1982	0.30905	0.08034
9. Secondary incomplete	0.52292	0.29959	0.75045	0.26142
10. Secondary complete	0.52199	0.4678	0.59016	0.4856
11. More than secondary	0.80864	0.99121	1.00338	0.82009
12. Vocational	0.95369	0.5123	0.26627	0.04142
**HH main source of income**				
13. Works for pay	0.29333	0.90787	0.5955	0.63684
14. Receives income from spouse/other members	0	0.89863	0.51623	0.69438
15. Unpaid on family business/farm	-0.08096	0	0	0
16. Unemployed	-0.0818	0.21042	0.49894	0.41075
17. Economically inactive	NA	NA	0.41792	-0.0899
**Housing quality**				
18. Persons per room	-0.03557	-0.05222	-0.05609	-0.13932
19. Owns land	0	0	0	0
20. Pays rent	0.45241	3.08815	0.41014	0.30171
21. No rent with owners consent	0.00688	1.50068	0.13849	-0.39251
22. Squatters	0.12971		0.0889	-0.65033
23. Owns bicycle	0.32374	0.1252	0.10902	0.14899
24. Owns motorcycle	0.97494	1.05757	0.24656	0.94915
25. Owns car or truck	0.71466	0.80907	1.23296	0.76588
26. Owns radio	0.28078	0.23943	0.19993	0.29401
27. Owns TV	0.80305	0.45529	1.03836	0.7559
28. Owns video	1.10786	1.26776	1.70521	1.10841
29. Owns refrigerator	1.51161	1.62935	2.04028	1.2577
30. Uses electricity	1.02303	1.76088	1.44804	NA
31. Uses solar power	1.12061	0.45853	1.09095	0.96362
32. Uses flush toilets	1.37463	1.28983	1.42158	1.04173
33. Uses pit latrines	0.43992	0.19288	0.41397	0.50787
34. Owns a phone	0.83599	0.48605	0.77271	0.67088
35. House has stone walls	1.60088	0	0.8332	0
36. House has clay walls	0	-1.61829	-0.63197	-1.20917
37. House has other type wall	1.15311	NA	-0.0602	-0.34895
38. House has stone floor	1.53363	1.62473	1.80454	1.28434
39. House has earth floor	0	0	0	0
40. House has other type of floor	0.89276	0.81848	1.1128	0.82777
41. House has tiled roof	1.10627	0.64139	1.01052	-0.07224
42. House has iron sheets roof	0.87779	0	1.10361	0
43. House has grass roof	0	-0.60852	0	-1.24814
44. Uses electricity, gas or kerosene	0.50503	1.89105	2.67614	0
45. Uses charcoal	1.0706	2.80889	3.17585	0.40986
46. Uses wood	0	0	0	-1.05246

* These are continuous variables. All other variables have a yes/no response

NA – refers to variables that were dropped due to zero variance.
